# Data-Driven Surgical Referrals for Primary Hyperparathyroidism and Associated Surgical Outcomes: A Simulation Study

**DOI:** 10.1245/s10434-025-17699-7

**Published:** 2025-07-22

**Authors:** David Rekhtman, Danielle E. Brown, Jasmine Hwang, James Sharpe, J. Walker Rosenthal, Caitlin Finn, Douglas Fraker, Rachel Kelz

**Affiliations:** 1 Perelman School of Medicine, University of Pennsylvania, Philadelphia, PA USA; 2 Division of Endocrine and Oncologic Surgery, Department of Surgery, Hospital of the University of Pennsylvania, Philadelphia, PA USA; 3 Center for Surgery and Healthcare Economics, Department of Surgery, Hospital of the University of Pennsylvania, Philadelphia, PA USA

**Keywords:** Primary hyperparathyroidism, Health system sciences, Simulation study, Parathyroidectomy

## Abstract

**Background:**

To minimize complications after parathyroidectomy, it is critical to connect patients with facilities equipped to perform this specialized procedure. This study assessed the effect of “referring” patients to higher-performing facilities for parathyroidectomy.

**Methods:**

A simulation study of adults who underwent parathyroidectomy for primary hyperparathyroidism was conducted using the Healthcare Cost and Utilization Project State Inpatient and Ambulatory Surgery and Services databases. Wilson score estimates were used to generate serious adverse event (SAE) rates for a training cohort to divide surgical facilities into quality quartiles. Using each facility’s fixed effect from the regression model, this study simulated the difference in SAE and cost for each patient between treatment at their original facility and treatment at an alternative higher-quality facility based on a lower SAE rate. The set of potential alternative facilities was determined based on proximity or original facility health system affiliation.

**Results:**

Of the 14,738 patients included in the proximity analysis 11,733 were randomized to the training group and 3005 to the testing cohort. The baseline characteristics and outcomes did not differ between the cohorts. Among the patients in the testing cohort, 314 were simulated to receive care at a higher-quality facility. The simulation predicted decreased SAE rates (2% vs. 3%; *p* < 0.001), with slightly increased total cost ($6391 vs. $6,351; *p* = 0.032). The results in the two simulations were similar.

**Conclusions:**

Simulation indicated that data-driven parathyroidectomy referrals can reduce SAE and advance surgical health equity. Data-driven facility selection is one way to achieve better surgical outcomes.

**Supplementary Information:**

The online version contains supplementary material available at 10.1245/s10434-025-17699-7.

Surgery has been the gold standard for definitive management of primary hyperparathyroidism (PHPT) since the 20th century. When performed successfully, parathyroidectomy offers significant and long-lasting improvement in clinical disease and quality of life.^1–4^ Despite the high volume of cases managed annually, a reported rise in reoperations after unsuccessful surgery has been reported.^5^ This trend may be due to a variety of reasons. First, earlier detection may result in smaller adenomas, which are more challenging to visualize perioperatively.^6^ Second, despite an increased reliance on technology, visualization remains poor because nearly 20% of patients with PHPT have negative imaging preoperatively.^7–12^ Third, increased centralization of surgical care, with increasing volume at previously higher-volume centers, has led to higher rates of reoperations at lower-volume centers.^5^

The association between surgical volume and outcomes has historically been used as a proxy metric for quality of surgical care. Complication and reoperation rates for parathyroidectomy are inversely related to surgeon volume and experience.^5^ As a result, the most recent guidelines for definitive management of PHPT from the American Association of Endocrine Surgeons recommend that parathyroidectomies be performed by surgeons with significant experience, which they define as an annual case rate of more than 10 operations per year.^13^ However, although surgical volume serves as an important proxy for quality, surgical outcomes are the ultimate measure of quality.

Simulation methodology gives us the ability to better understand the impact of optimizing care patterns based on outcomes rather than using volume as a proxy. Measurement of a facility’s serious adverse event (SAE) and reoperation rate allows for an assessment of a facility’s quality with regard to performing parathyroidectomies for PHPT.

In this study, we used validated methodology to determine risk-adjusted facility performance.^14,15^ We then performed a simulation study in which adults were referred to higher-performing local facilities and evaluated the difference in outcomes. We hypothesized that optimized referral patterns would result in decreased SAE, cost, and need for reoperation.

## Methods

### Patient Selection

We performed a simulation study using the Healthcare Cost and Utilization Project State Inpatient and Ambulatory Surgery and Services databases from Florida, Maryland, Vermont, Utah, and Wisconsin. These states were included because their datasets allow for linkage of different encounters at various facilities and time points to the same patient, which enables identification of patients with rehospitalization and reoperations at alternative facilities from their index operation. International Classification of Disease Tenth Revision (ICD-10) procedure codes and Current Procedural Terminology (CPT) codes were used to identify adult patients who underwent parathyroidectomy for PHPT between 1 January 2016 and 31 December 2020.

The inclusion and exclusion criteria are listed in Table S1. The study excluded patients who lived outside the state in which they received care and those who had undergone any endocrine surgery in the neck during the 6 months before their parathyroidectomy. Additionally, facilities with fewer than 10 cases per year were excluded from the simulation as a result of unstable estimates due to large standard errors. Despite exclusion from the main analysis, a secondary analysis, inclusive of the lowest-volume centers, was performed to assess with relationship between Wilson score estimates and facility volume and reported SAE rate.

**Fig. 1:**
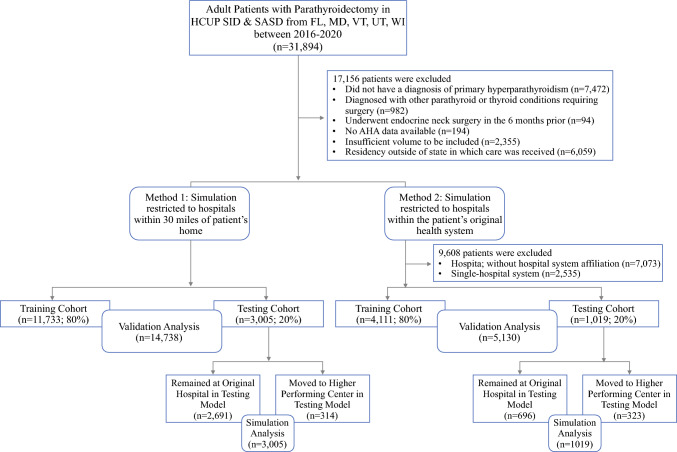
Consort diagram. The study’s inclusion and exclusion criteria are depicted as well as subsequent analyses using training and testing cohorts

Two simulation methods were developed to determine the impact of assigning patients to “higher-performing” facilities on postoperative outcomes (Fig. 1). All facilities were ranked and then divided into quartiles based on cumulative SAE burden. In “Method 1,” patients were restricted to facilities within 30 miles of their home zip code, whereas in “Method 2,” patients could be simulated to any alternative facility within the original facility’s health system regardless of distance. Health system status was determined based on a unique identifier in the American Hospital Association (AHA) database. To perform the “Method 2” simulation, patients who had received care from a facility without a system affiliation or a single-facility system were excluded before the development of training and testing cohorts. For both methods, 80% of patients were randomly assigned to the training cohort, with the remaining 20% selected for the testing cohort.

### Facility Quality Determination from the Training Cohort

The patients in the training cohort were used to calculate a risk-standardized 30 day SAE rate. This composite metric included technical events after parathyroidectomy, medical postoperative events, 30 day urgent or emergent readmission, and death. Technical events included vocal cord dysfunction, hypocalcemia, tracheostomy, bleeding, and surgical-site infection. Medical events included atrial fibrillation, cardiac event, cerebrovascular complication, renal infection, renal dysfunction, respiratory complication, sepsis, transient ischemic attack, and venous thromboembolism. All events were identified based on ICD or CPT codes as documented within 30 days of the index operation but not present at the time of the operation (Table S2).

To account for facility volume variability, Wilson score intervals were used to estimate the true SAE rate. This statistical methodology provides a confidence interval for each proportion that is dependent on the procedure sample size and reported SAE rate of the facility.^16,17^ This approach has particular utility for facilities in which the SAE rate is near 0 or 1 because these are also facilities in which traditional methods, such as predictive logistic regression models, struggle to produce reliable estimates.^16,17^

From the Wilson score confidence interval, the midpoint was calculated. Facilities were then ranked based on their respective Wilson score midpoint and divided into quartiles based on quality.

### Model Design and Simulation Strategy

To assess for bias in cohort selection, baseline characteristics including age, sex, race, insurance, type, symptomatology, surgery setting, parathyroidectomy volume, patient-facility distance, and Elixhauser index comorbidities were compared between the training and testing groups. Additionally, unadjusted outcomes such as hospital length of stay, SAE rates, technical events, medical events, readmission at 30 days, and reoperation within 2 years were compared. Total cost also was assessed. Cost was calculated by multiplying the total charges by the cost-to-charge ratio, adjusting for the wage index, and then converting all costs to 2020 U.S. dollars using the Consumer Price Index for medical care.^18^

The testing cohort then was used to evaluate the impact of referral patterns on patient outcomes. Patients who had an alternative facility in a higher-quality quartile were simulated to receive their parathyroidectomy there. The simulations considered only facilities within a 30 mile radius of the patient’s home zip code (Method 1) or within the original facility’s health system (Method 2). The assumption was made that patients who underwent parathyroidectomy at a lower-performing facility would be willing to receive operative treatment at the higher-performing facility.

The baseline characteristics of the patients who remained at their original facility were compared with those of the patients simulated to receive care at an alternative higher-quality center. We then simulated the difference in SAE and cost for each patient. 

### Statistical Analysis

All continuous nonparametric variables were assessed using a generalized Wilcoxon rank-sum test, whereas categorical variables were compared using a chi-square test. Fisher’s exact test was used for categorical variables with low expected frequencies. When baseline characteristics after simulation were compared, a paired analysis was performed for variables not inherent to the patient such as facility volume or patient-facility distance. All statistical analyses were considered significant at a two-sided significance level of 0.05. 

In the simulation, a patient’s SAE risk was predicted by using the facility’s fixed effect from the regression. The paired *t* test and the Wilcoxon signed-rank test were used to compare outcomes obtained from applying the simulation models to the original 20% testing cohort with the predicted values from the same testing cohort after reception of care at a higher-quality facility. The reported outcome is therefore the relative difference in SAE risk between a patient’s original and alternative higher-quality facility.

Hospital length of stay was modeled using Poisson regression. Cost was modeled using linear regression, and all other outcomes were modeled with logistic regression. Control was used for the following covariates in all the models: patient age, race, sex, insurance, setting (in- or outpatient), admission type (emergent/urgent or elective), year of discharge, facility volume (as tertiles), patient-facility distance (miles between the center of the patient’s zip code and the facility’s zip code), Elixhauser index comorbidities, and fixed effects for facility.

Statistical analysis was performed using SAS, version 9.4 (SAS Institute, Cary, NC, USA), whereas R 4.0.2 (R Foundation for Statistical Computing, Vienna, Austria) was used for advanced modeling. This study was deemed exempt by the Institutional Review Board at the University of Pennsylvania (protocol #852123).

## Results

### Patient Characteristics

Of the 31,894 patients who underwent parathyroidectomy during the study period, 14,738 met the inclusion criteria. The median age of this population was 65 years (interquartile interval [IQI], 56–72 years). The majority were female (77%), were white (78%), and had either Medicare (50%) or private (44%) insurance. The vast majority of cases (97%) were managed in an outpatient ambulatory surgery center with a median parathyroidectomy volume of 154 cases (IQI, 55–3173 cases) per facility per year. The relationship between the Wilson score estimates and the reported SAE rate (Fig. S1) as well as the facility volume (Fig. S2) was graphically assessed. A secondary analysis that included facilities with fewer than 10 cases per year (Fig. S3) demonstrated similar trends, suggesting that the results would extend to these centers as well.

### Method 1: Simulation of Alternative Facility Selection Based on Proximity to Patient

After randomization along an 80/20 split, 11,733 patients were included in the training cohort, with the remaining 3005 patients assigned to the testing cohort. No difference was seen in baseline characteristics (Table 1) or outcomes (Table 2) between the training and testing groups. Facility quality was determined based on Wilson score estimates of the SAE rate in the training cohort. The median SAE rate was found to be 7% (IQI, 4–13%).

**Table 1:** Baseline characteristics of patients included in this study^a^

	Method 1: Proximity analysis			Method 2: Facility system analysis		
Variable	Training cohort (*n* = 11,733) *n* (%)	Testing cohort (*n* = 3005) *n* (%)	*p* value	Training cohort (*n* = 4131) *n* (%)	Testing cohort (*n* = 1034) *n* (%)	*p* Value
Median age: years (IQI)	65 (56–72)	65 (56–71)	0.814	63 (54–71)	64 (55–71)	0.715
Female sex	9038 (77.0)	2310 (76.9)	0.873	3208 (77.7)	796 (77.0)	0.673
Race			0.114			0.269
Black	965 (8.2)	235 (7.8)		473 (11.5)	111 (10.7)	
Hispanic	898 (7.7)	271 (9.0)		481 (11.6)	146 (14.1)	
White	9121 (77.7)	2319 (77.2)		2778 (67.3)	685 (66.3)	
Other	234 (2.0)	62 (2.1)		85 (2.1)	20 (1.9)	
Missing	515 (4.4)	118 (3.9)		314 (7.6)	72 (7.0)	
Insurance			0.965			0.488
Medicaid	274 (2.3)	74 (2.5)		132 (3.2)	34 (3.3)	
Medicare	5909 (50.4)	1519 (50.5)		1911 (46.3)	504 (48.7)	
Private	5216 (44.5)	1325 (44.1)		1957 (47.4)	462 (44.7)	
Other or missing	334 (2.8)	87 (2.9)		131 (3.2)	34 (3.3)	
Admission type			0.216			0.707
Elective	3526 (30.1)	856 (28.5)		1667 (40.4)	418 (40.4)	
Emergent/urgent	158 (1.3)	45 (1.5)		94 (2.3)	28 (2.7)	
Other or missing	8049 (68.6)	2104 (70.0)		2370 (57.4)	588 (56.9)	
Symptoms						
Fracture	–	0 (0.0)	0.609	–	0 (0.0)	1.000
Osteoporosis	1098 (9.4)	273 (9.1)	0.671	431 (10.4)	117 (11.3)	0.443
Nephrolithiasis	114 (1.0)	18 (0.6)	0.068	63 (1.5)	12 (1.2)	0.465
Hypercalciuria	–	–	1.000	–	–	1.000
Admission setting			1.000			0.532
Ambulatory	11,361 (96.8)	2910 (96.8)		3891 (94.2)	968 (93.6)	
In-patient	372 (3.2)	95 (3.2)		240 (5.8)	66 (6.4)	
Median facility volume (IQI)	154 (555–3173)	154 (55–3173)	0.700	61 (25–93)	58 (26–90)	0.281
Patient-to-facility Median distance: miles (IQI)	19 (7–63)	19 (8–67)	0.483	9 (5–19)	9 (5–18)	0.369
Elixhauser categories			0.715			0.546
0	3670 (31.3)	917 (30.5)		1,239 (30.0)	293 (28.3)	
1	3451 (29.4)	888 (29.6)		1120 (27.1)	281 (27.2)	
2	2565 (21.9)	683 (22.7)		897 (21.7)	244 (23.6)	
3+	2047 (17.4)	517 (17.2)		875 (21.2)	216 (20.9)	

*IQI* interquartile interval

^a^ Categorical data are expressed as *n* (%), whereas continuous data are expressed as median (IQI). Bold type denotes *p* < 0.05. If the “missing” or “other” category had <10 patients, the two categories were combined. “–“ indicates a prevalence of <11 in a variable as required by the data-use agreement

**Table 2:** Baseline unadjusted outcomes for the patients included in this study^a^

	Method 1: Proximity analysis			Method 2: Facility system analysis		
Variable	Training cohort (*n* = 11,733) *n* (%)	Testing cohort (*n* = 3005) *n* (%)	*p* Value	Training cohort (*n* = 4131) *n* (%)	Testing cohort (*n* = 1034) *n* (%)	*p* Value
Median length of stay: days (IQI)	0 (0–0)	0 (0–0)	0.414	0 (0–1)	0 (0–1)	0.302
Routine disposition	11,170 (95.2)	2850 (94.8)	0.526	3899 (94.4)	980 (94.8)	0.675
Median total cost: 2020: USD (IQI)	6269 (5334–7595)	6263 (5339–7524)	0.656	6220 (4969–7827)	6305 (4930–7797)	0.710
30-Day serious adverse event	298 (2.6)	89 (3.1)	0.217	148 (3.7)	42 (4.2)	0.524
Reoperation within 2 years	104 (0.9)	30 (1.0)	0.639	49 (1.2)	12 (1.2)	1.000
Technical events at 30-days	92 (0.8)	30 (1.0)	0.297	35 (0.8)	12 (1.2)	0.444
Vocal cord dysfunction	–	–	0.119	–	–	0.056
Hypocalcemia	67 (0.6)	18 (0.6)	0.964	–	–	0.963
Tracheostomy	–	0 (0.0)	1.000	0 (0.0)	0 (0.0)	1.000
Bleeding/hematoma	–	–	1.000	–	–	0.633
Medical events at 30-days	235 (2.0)	70 (2.3)	0.294	128 (3.1)	38 (3.7)	0.400
Atrial fibrillation	59 (1.7)	19 (2.2)	0.385	–	–	1.000
Cardiac Event	153 (1.3)	41 (1.4)	0.866	89 (2.2)	19 (1.8)	0.606
Cerebrovascular Complication	–	–	**0.029**	–	–	**0.010**
Renal infection	–	0 (0.0)	1.000	–	0 (0.0)	1.000
Renal dysfunction	44 (0.4)	14 (0.5)	0.585	–	–	0.716
Respiratory Complication	–	0 (0.0)	1.000	–	0 (0.0)	1.000
Sepsis	–	–	0.488	–	0 (0.0)	0.219
Venous Thromboembolism	–	–	0.354	0 (0.0)	0 (0.0)	1.000
30-Day readmission	723 (6.2)	182 (6.1)	0.863	219 (5.3)	56 (5.4)	0.945

*IQI* interquartile interval, *USD* United States dollars

^a^ Categorical data are expressed as *n* (%), whereas continuous data are expressed as median (IQI). Bold type denotes *p* < 0.05. “–“ indicates a prevalence of <11 in a variable as required by the data-use agreement

Of the 3005 patients in the testing cohort, 314 (10%) were found to have a higher performing facility within 30 miles, allowing for simulation. The baseline characteristics for these two groups are described in Table 3. The cohort simulated to switch facilities was found to be similar to those who remained at their original hospital with regard to age (median, 63 vs. 65; *p* = 0.81) and sex (81% vs. 76% female; *p* = 0.064), but was more likely to be non-white (19% vs. 17%; *p* = 0.038) and to have Medicaid insurance (5% vs. 2%; *p* = 0.003). The patients eligible for a higher-quality facility had more comorbidities (50% vs. 39% had 2+ Elixhauser categories; *p* < 0.001). Patients simulated to move were “sent” to facilities with higher volumes (median, 44 vs. 31 cases/facility/year; *p* < 0.001) and were within a similar distance from their homes (median, 9 vs. 10 miles; *p* = 0.213).

**Table 3:** Simulation testing cohort characteristics of those who remained at original facility and those moved in simulation to a higher-performing facility^a^

	Method 1: Proximity analysis			Method 2: Facility system analysis		
Variable	Original (*n* = 2691) *n* (%)	Simulated (*n* = 314) *n* (%)	*p* Value	Original (*n* = 696) *n* (%)	Simulated (*n* = 323) *n* (%)	*p* Value
Median age: years (IQI)	65 (56–71)	63 (54–71)	0.081	64 (55–71)	63 (54–70)	0.507
Female sex	2055 (76.4)	255 (81.2)	0.064	529 (76.0)	254 (78.6)	0.397
Race			**0.038**			0.182
Black	199 (7.4)	36 (11.5)		80 (11.5)	33 (10.2)	
Hispanic	249 (9.3)	22 (7.0)		108 (15.5)	35 (10.8)	
White	2085 (77.5)	234 (74.5)		447 (64.2)	223 (69.0)	
Other or missing	158 (5.9)	22 (7.0)		61 (8.8)	32 (9.9)	
Insurance			**0.003**			0.155
Medicaid	57 (2.1)	17 (5.4)		15 (2.2)	15 (4.6)	
Medicare	1373 (51.0)	146 (46.5)		345 (49.6)	149 (46.1)	
Private	<1196 (<44.4)	>129 (>41.1)		314 (45.1)	148 (45.8)	
Other or missing	>76 (>2.8)	<11 (<3.5)		22 (3.2)	11 (3.4)	
Admission type			**0.001**			**<0.001**
Elective	636 (23.6)	220 (70.1)		212 (30.5)	198 (61.3)	
Emergent/urgent	>35 (>1.3)	<11 (<3.5)		>16 (>2.3)	<11 (<3.4)	
Other or missing	<2021 (<75.1)	>83 (>26.4)		<484 (<69.5)	>114 (>35.2)	
Symptoms						
Osteoporosis	229 (8.5)	44 (14.0)	**0.002**	80 (11.5)	32 (9.9)	0.518
Nephrolithiasis	–	–	0.425	–	–	0.060
Hypercalciuria	–	–	0.198	0 (0)	–	0.317
Admission setting			**0.001**			0.068
Ambulatory	2616 (97.2)	294 (93.6)		660 (94.8)	296 (91.6)	
In-patient	75 (2.8)	20 (6.4)		36 (5.2)	27 (8.4)	
Elixhauser Categories			**<0.001**			**0.020**
0	848 (31.5)	69 (22.0)		216 (31.0)	75 (23.2)	
1	799 (29.7)	89 (28.3)		191 (27.4)	83 (25.7)	
2	604 (22.5)	79 (25.2)		155 (22.3)	84 (26.0)	
3+	440 (16.4)	77 (24.5)		137 (19.3)	81 (25.1)	
	Original conditions (*n* = 314)	Simulated conditions (*n* = 314)		Original conditions (*n* = 323)	Simulated conditions (*n* = 323)	
Median facility volume (IQI)	31 (14–67)	44 (17–195)	**<0.001**	29 (14–63)	34 (15–78)	**<0.001**
Median patient-to-facility distance: miles (IQI)	9 (4–16)	10 (6–17)	0.213	9 (5–20)	25 (11–56)	**<0.001**

*IQI* interquartile interval

^a^ Categorical data are expressed as *n* (%), whereas continuous data are expressed as median (interquartile interval). For variables that changed with the simulation, a paired analysis was performed. Bold type denotes *p* < 0.05. If the “missing” or “other” category had <10 patients, the two categories were combined. “–“ indicates a combined prevalence of <11 in a variable as required by the data-use agreement

**Fig. 2:**
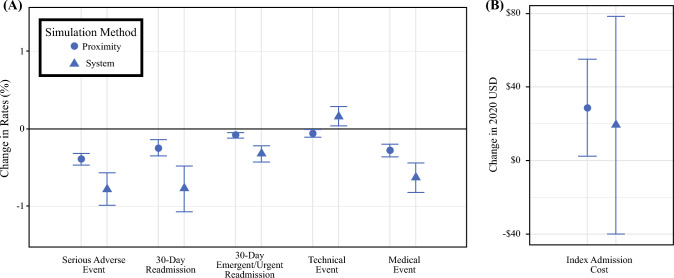
Predicted difference in **A** postoperative outcomes and **B** cost at alternative facilities. Method 1 (within 30 miles) is presented as circles, whereas Method 2 (within facility’s health system) is presented as triangles. Symbols represent point estimates with 95% confidence intervals

Paired analyses of the testing cohort before and after simulation predicted a slightly higher cost (mean, $6391 vs. $6351; *p* = 0.032) despite a shorter hospital length of stay (mean, 0.08 vs. 0.09 days; *p* = 0.001; Fig. 2B). Notably, parathyroidectomies performed at the simulated higher-quality facility resulted in lower rates of readmission (mean, 5% vs. 6%; *p* < 0.001), SAE (mean, 2% vs. 3%; *p* < 0.001), technical events (mean, 0.67% vs. 0.70%; *p* = 0.014), and medical events (mean, 1.7% vs. 1.9%; *p* < 0.001) (Fig. 2A).

### Method 2: Simulation of Alternative Facility Selection Based on Health System Affiliation

To perform the health system simulation, the study removed the patients who received their parathyroidectomy at a facility without a system affiliation or at a single-facility system. This resulted in a cohort of 5130 patients. Of these patients, 4111 (80%) were in the training cohort, and the remaining 1019 (20%) were in the testing cohort. As with the first model, no differences in baseline characteristics (Table 1) or outcomes were noted (Table 2).

Approximately 32% of the patients in the testing cohort (*n* = 323) were simulated to a higher-performing center in the testing model. Age, sex, race, and insurance status did not differ between the group that was simulated to receive a parathyroidectomy at a different center and the group that was not (all *p* > 0.05) (Table 3). However, the patients simulated to move had more comorbidities than those simulated to remain in their original facility (median: 51% vs. 42% had 2+ Elixhauser categories; *p* = 0.020). On the average, the simulation moved patients to a further facility (median, 25 vs. 9 miles; *p* < 0.001) that had a higher annual parathyroidectomy volume (median, 34 vs. 29 cases/year; *p* < 0.001).

Restricting the simulation of patients to higher-performing facilities within the same health system that managed their original operations resulted in similarly decreased SAE rates (mean, 3% vs. 4%; *p* < 0.001), medical events (mean, 2% vs. 3%; *p* < 0.001), and 30 day readmissions (mean, 4% vs. 5%; *p* < 0.001) as the simulation restricted by travel distance. The analysis restricted by system affiliation showed a slight (likely clinically insignificant) increase in hospital length of stay (mean, 0.7 vs. 0.6 days; *p* < 0.001) without a significant difference in cost (mean, $6860 vs. $6920; *p* = 0.524).

## Discussion

Our study simulated how data-driven referral patterns for patients with PHPT requiring parathyroidectomy could impact surgical outcomes. Both methods of simulation showed a reduced rate of SAE and medical events when patients were sent to higher-performing facilities for care despite increased comorbidities in the patient population. Cost was statistically higher, but not meaningfully different, when patient referrals were permitted across facilities regardless of health system affiliations but restricted by travel distance. No differences in cost were observed when referrals were restricted to facilities within the same health system. Taken together, data-driven surgical referrals at both the regional and health system level may improve outcomes for parathyroidectomy.

Referral patterns for parathyroidectomy have varied over time as the practice of medicine has evolved. The 21st century has shown a shift toward performance of parathyroidectomy for PHPT within an ambulatory setting, with the majority of operations currently performed without inpatient stays.^19,20^ Patients typically are sent to a surgeon by their primary care physician, but may be referred by a specialist such as an endocrinologist.^21,22^ Among surveyed endocrinologists, factors that determined their surgeon of choice included the surgeon’s outcomes, volume, and communication style, both with the referring provider and previous patients.^23^

Our study highlights the utility of simulation studies to inform referral practices based on facility quality with regard to a highly specialized operation. The findings show a significant reduction in SAE, technical events, and medical events when referrals are based on risk-adjusted facility quality measured by past performance.

Guidelines clearly demonstrate that surgical management of PHPT is indicated for symptomatic patients and is a cost-effective intervention.^24,25^ Parathyroidectomy is both less costly than pharmacologic therapies and more effective in improving quality of life for patients experiencing symptoms of hypercalcemia.^24^ Additionally, it also has been reported that the cost of parathyroidectomy varies greatly based on a surgeon’s practice and the facility’s structure.^26^ Jang et al.^26^ described their institution’s experience and noted a wide range of costs, from $4522.30 to $12,072.87, depending on the provider. The median cost reported in our analyses was well within this range. The minimally increased cost of $40 associated with referral to higher-quality centers may stem from the demographics of the patients selected for data-driven referrals in the simulation. For instance, findings have shown black to be associated with a higher cost for parathyroidectomy.^27^ This difference may result from the disparities in access to timely intervention as well as from comorbidities that these patients have due to structural and social determinants. Regardless, the increased facility costs are minimal and may be negated by the improved outcomes and reduced rates of complications and readmissions.

This study had certain limitations. First, the study was restricted to the analysis of patients within five states, which may not be fully representative of the nation.

Second, facilities with low volume have unreliable SAE rates. To overcome this, we used Wilson score estimates to develop quality quartiles, and in so doing brought additional rigor to the certainty of our estimates. Although Wilson score estimates allowed for the inclusion of facilities with low surgical volumes, we still were required to exclude facilities with fewer than 10 cases per year due to unstable estimates, although these centers would likely have the greatest benefit from this type of simulation study.

Third, our simulation identified the highest-quality facility solely based on SAE rates. We did not factor in patient preferences, provider experiences, referral practices, or limitations of individual patients’ insurance plans.

Furthermore, implementation of this approach would have required patient-centered discussions regarding the tradeoffs between a more proximate center and one with a higher likelihood of an improved clinical outcome. Given the heterogeneity of patients, the proposed changes in referrals may not be possible and should be viewed as an idealistic possibility. Further studies using more granular data should focus on the feasibility of optimizing referrals based on patient-specific limitations.

This simulation study demonstrated that higher-quality facilities are available to patients within a reasonable geographic region, which can lead to improved outcomes as measured by reduced medical events, technical events, and reoperation rates. Furthermore, optimizing referral practices does not have a significant impact on the financial cost to the institution while allowing patients to receive higher-quality care.

## Disclosure

There are no conflicts of interest.

## Supplementary Information

Below is the link to the electronic supplementary material.

Supplementary file1 (DOCX 212 kb)
