# Supplementary material for: Data-Driven Surgical Referrals for Primary Hyperparathyroidism and Associated Surgical Outcomes: A Simulation Study
Source: Ann Surg Oncol. 2025 Jul 22;32(10):7489–97. doi: 10.1245/s10434-025-17699-7 (PMC12454539; doi:10.1245/s10434-025-17699-7)
Supplement: Supplementary file 1 — Supplementary file1 (DOCX 212 kb) [file 10434_2025_17699_MOESM1_ESM.docx]

**SUPPLEMENTAL**

**Supplemental Table 1: ICD-10 and CPT Codes associated with inclusion and exclusion criteria.**

| **Name** | **ICD-10 Code** | **CPT Code** |
| --- | --- | --- |
| **Inclusion Criteria** |  |  |
| Parathyroidectomy | 0GTL0ZZ, 0GTL4ZZ, 0GTM0ZZ, 0GTM4ZZ, 0GTN0ZZ, 0GTN4ZZ, 0GTP0ZZ, 0GTP4ZZ, 0GTQ0ZZ, 0GTQ4ZZ, 0GTR0ZZ, 0GTR4ZZ, 0GBL0ZX, 0GBL0ZZ, 0GBL4ZX, 0GBL4ZZ, 0GBM0ZX, 0GBM0ZZ, 0GBM4ZX, 0GBM4ZZ, 0GBN0ZX, 0GBN0ZZ, 0GBN4ZX, 0GBN4ZZ, 0GBP0ZX, 0GBP0ZZ, 0GBP4ZX, 0GBP4ZZ, 0GBQ0ZX, 0GBQ0ZZ, 0GBQ4ZX, 0GBQ4ZZ, 0GBR0ZX, 0GBR0ZZ, 0GBR4ZX, 0GBR4ZZ, 0GCL0ZZ, 0GCL3ZZ, 0GCL4ZZ, 0GCM0ZZ, 0GCM3ZZ, 0GCM4ZZ, 0GCN0ZZ, 0GCN3ZZ, 0GCN4ZZ, 0GCP0ZZ, 0GCP3ZZ, 0GCP4ZZ, 0GCQ0ZZ, 0GCQ3ZZ, 0GCQ4ZZ, 0GCR0ZZ, 0GCR3ZZ, 0GCR4ZZ | 60500, 60505 |
| Primary Hyperparathyroidism | E210 |  |
| **Exclusion Criteria** |  |  |
| Secondary hyperparathyroidism | N2581, E211 |  |
| Tertiary hyperparathyroidism | Z94.0, Z48.22, T86.10, T86.11, T86.12, T86.13, T86.19, Z79.52, Z79.60, Z79.61, Z79.621, Z79.622, Z79.623, Z79.624, Z79.631, Z79.69, Z92.25 |  |
| Parathyroid Cancer | C750 |  |
| Multiple Endocrine Neoplasia (MEN) Syndrome | E31.2x |  |
| Dialysis Dependence | N18.6, Z99.2, N19, Z49.01, Z49.02, Z94.15, Z49.31, Z49.32, I12.0, I13.11, I13.2 |  |
| Re-operation |  | 60502 |
| Partial Thyroidectomy | 0GBG0ZX, 0GBG0ZZ, 0GBG4ZX, 0GBG4ZZ, 0GBH0ZX, 0GBH0ZZ, 0GBH4ZX, 0GBH4ZZ, 0GBJ0ZX, 0GBJ0ZZ, 0GBJ4ZX, 0GBJ4ZZ, 0GCG0ZZ, 0GCG4ZZ, 0GCH0ZZ, 0GCH4ZZ, 0GTG0ZZ, 0GTG4ZZ, 0GTH0ZZ, 0GTH4ZZ, 0GTJ0ZZ, 0GTJ4ZZ, 0GTJ4ZZ | 60200, 60210, 60212, 60220, 60225, 60245, 60246 |
| Total Thyroidectomy | 0GCK0ZZ, 0GCK4ZZ, 0GTK0ZZ, 0GTK4ZZ | 60270, 60271, 60225, 60240, 60260, 60270, 60271 |
| Total Thyroidectomy with Neck Dissection | 07B10ZX, 07B10ZZ, 07B13ZX, 07B13ZZ, 07B14ZX, 07B14ZZ, 07B20ZX, 07B20ZZ, 07B23ZX, 07B23ZZ, 07B24ZX, 07B24ZZ, 07T10ZZ, 07T14ZZ, 07T20ZZ, 07T24ZZ | 60252, 60254, 38700, 38724, 38746, 38720 |
| **Pre-operative Symptoms** |  |  |
| Fracture | S02.x, S12.x, S22.x, S32.x, S42.x, S52.x, S62.x, S72.x, S82.x, S92.x, T02.x, T08.x, T10.x, T12.x, T14.2x, M48.5x |  |
| Osteoporosis | M80.x, M81.x |  |
| Nephrolithiasis | N20.x |  |
| Hypercalciuria | R82.994 |  |

Lower case “x” indicates all valid digits that can complete the code. ICD = International Classification of Diseases; CPT = Current Procedural Terminology; PCS = Procedure Coding System for inpatient setting; CM = Clinical Modification for outpatient setting.

**Supplemental Table 2: ICD-10 and CPT codes associated with post-operative events.**

| **Name** | **ICD-10 Code** | | **CPT Code** |
| --- | --- | --- | --- |
| **Post-operative Technical Events** |  | |  |
| Vocal Cord Dysfunction | J3800, J3801, J3802, J383, R490, R491, R498, R499, G9781, G9782, G522 | |  |
| Hypocalcemia | E8351, E892, E200, E208, E209, R290 | | J0610, J0620 |
| Tracheostomy | ICD-10-CM: Z930 | ICD-10-PCS: 0B110F4, 0B110Z4, 0B113F4, 0B113Z4, 0B114F4, 0B114Z4 | 31600, 31603, 31605, 31610 |
| Bleeding/Hematoma | L7602, L7622, L7632, E8981, E89810, E89811, E89820, E89821 | |  |
| Surgical Site Infection | T8130, T8131, T8132, T8140, T8141, T8142, T8143, T8144, T8149 | |  |
| **Post-operative Medical Events** |  | |  |
| Atrial Fibrillation | I48.91, I97.710, I97.790, I97.88, I97.89 | |  |
| Cardiac Events | I01.0, I01.8, I02.0, I11.0, I13.0, I13.2, I20.0, I21.09, I21.11, I21.19, I21.29, I21.3, I21.4, I23.0, I24.0, I24.1, I24.8, I25.3, I25.41, I25.42, I30.0, I30.8, I30.9, I31.4, I32, I44.0, I44.1, I44.2, I44.30, I45.81, I45.89, I46.9, I47.1, I47.2, I49.01, I49.02, I49.1, I49.3, I49.40, I49.49, I49.5, I49.8, I49.9, I50.1, I50.20, I50.21, I50.22, I50.23, I50.30, I50.31, I50.32, I50.33, I50.40, I50.41, I50.42, I50.43, I50.9, I51.0, I51.2, J81.0, R00.1, R57.0, R57.9, R94.30, R94.31, T82.110A, T82.111A, T82.120A, T82.121A, T82.190A, T82.191A | |  |
| Cerebrovascular Complications | G03.8, G45.4, G97.0, G97.81, G97.82, I60.9, I61.9, I62.00, I62.1, I62.9, I63.019, I63.119, I63.139, I63.20, I63.219, I63.22, I63.239, I63.30, I63.40, I63.50, I63.59, I65.09, I65.1, I65.29, I65.8, I65.9, I66.09, I66.19, I66.29, I66.9, I67.1, I67.2, I67.4, I67.5, I67.6, I67.7, I67.81, I67.82, I67.89, I67.9, I69.898, I69.90, I69.91, I69.920, I69.921, I69.922, I69.923, I69.928, I69.931, I69.932, I69.933, I69.934, I69.939, I69.941, I69.942, I69.943, I69.944, I69.949, I69.951, I69.952, I69.953, I69.954, I69.959, I69.961, I69.962, I69.963, I69.964, I69.965, I69.969, I69.990, I69.991, I69.992, I69.993, I69.998, I97.811, I97.821 | |  |
| Renal Infection | N10, N12, N15.1, N15.9, N16, N28.84, N28.85, N28.86 | |  |
| Renal Dysfunction | N17.0, N17.1, N17.2, N17.8, N17.9, R34, R88.0, T82.818A, T85.71XA, Y62.2, Y84.1, Z49.01, Z49.02, Z49.31, Z49.32, Z99.2 | |  |
| Respiratory Complication | J98.01, R09.2 | |  |
| Sepsis | A40.3, A40.9, A41.01, A41.02, A41.1, A41.2, A41.3, A41.4, A41.50, A41.51, A41.52, A41.53, A41.59, A41.89, A41.9, I76, R78.81 | |  |
| Transient Ischemic Attack | G45.0, G45.1, G45.8, G45.9, I67.848 | |  |
| Venous Thromboembolism | I26.90, I26.92, I26.99, I82.290, I82.409, I82.419, I82.429, I82.439, I82.449, I82.499, I82.4Y9, I82.4Z9, I82.609, I82.619, I82.629, I82.890, I82.91, I82.A19, I82.B19, I82.C19, T79.1XXA, T80.0XXA, T81.718A, T81.72XA, T82.817A | |  |
| **Re-operation** | 0GTL0ZZ, 0GTL4ZZ, 0GTM0ZZ, 0GTM4ZZ, 0GTN0ZZ, 0GTN4ZZ, 0GTP0ZZ, 0GTP4ZZ, 0GTQ0ZZ, 0GTQ4ZZ, 0GTR0ZZ, 0GTR4ZZ, 0GBL0ZX, 0GBL0ZZ, 0GBL4ZX, 0GBL4ZZ, 0GBM0ZX, 0GBM0ZZ, 0GBM4ZX, 0GBM4ZZ, 0GBN0ZX, 0GBN0ZZ, 0GBN4ZX, 0GBN4ZZ, 0GBP0ZX, 0GBP0ZZ, 0GBP4ZX, 0GBP4ZZ, 0GBQ0ZX, 0GBQ0ZZ, 0GBQ4ZX, 0GBQ4ZZ, 0GBR0ZX, 0GBR0ZZ, 0GBR4ZX, 0GBR4ZZ, 0GCL0ZZ, 0GCL3ZZ, 0GCL4ZZ, 0GCM0ZZ, 0GCM3ZZ, 0GCM4ZZ, 0GCN0ZZ, 0GCN3ZZ, 0GCN4ZZ, 0GCP0ZZ, 0GCP3ZZ, 0GCP4ZZ, 0GCQ0ZZ, 0GCQ3ZZ, 0GCQ4ZZ, 0GCR0ZZ, 0GCR3ZZ, 0GCR4ZZ | | 60500, 60502, 60505 |

Lower case “x” indicates all valid digits that can complete the code. ICD = International Classification of Diseases; CPT = Current Procedural Terminology; PCS = Procedure Coding System for inpatient setting; CM = Clinical Modification for outpatient setting.

**Supplemental Figure 1: Wilson Score Estimate Compared to Reported Facility Serious Adverse Event Rate.**


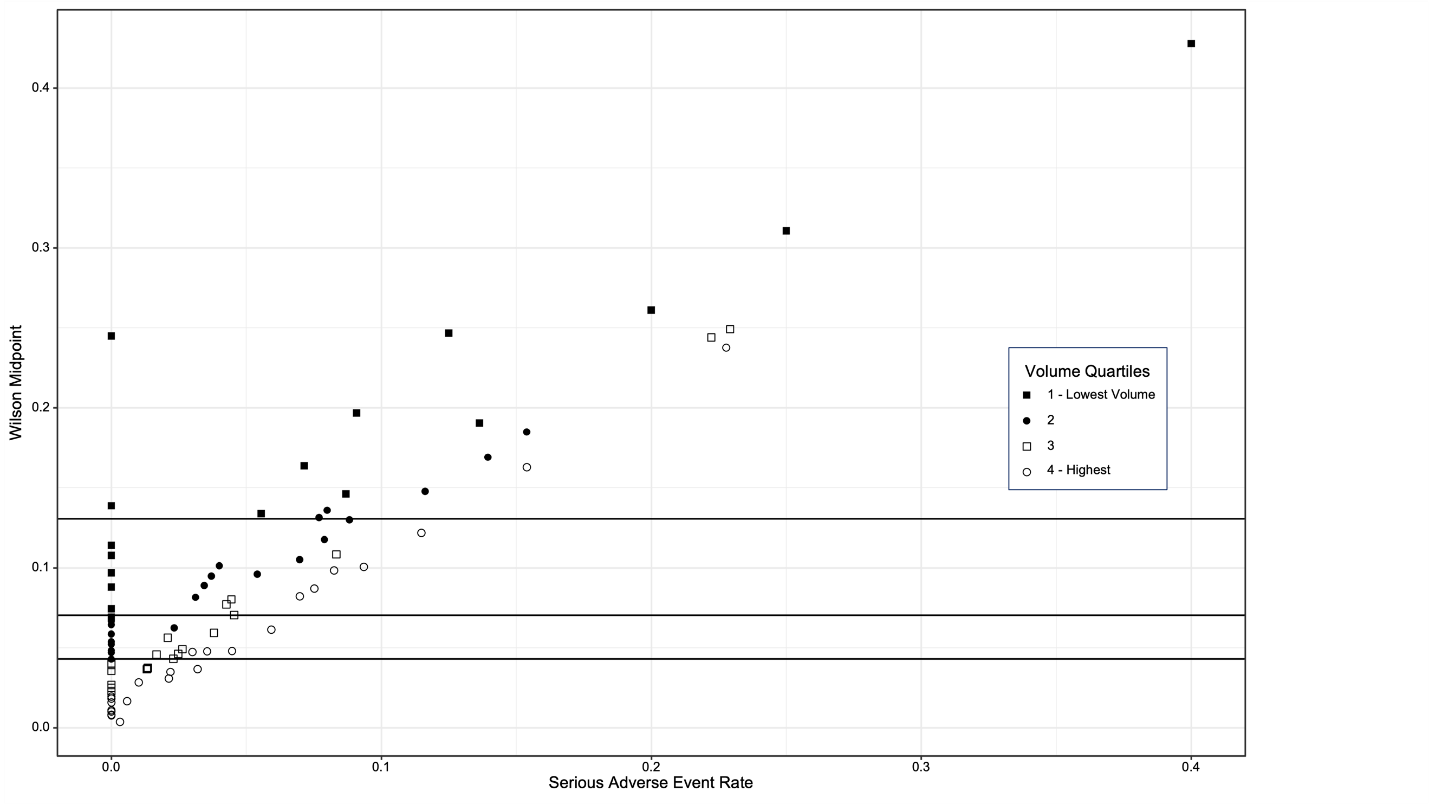


**Supplemental Figure 2: Relationship between Wilson Score Estimate and Facility Volume.**

**
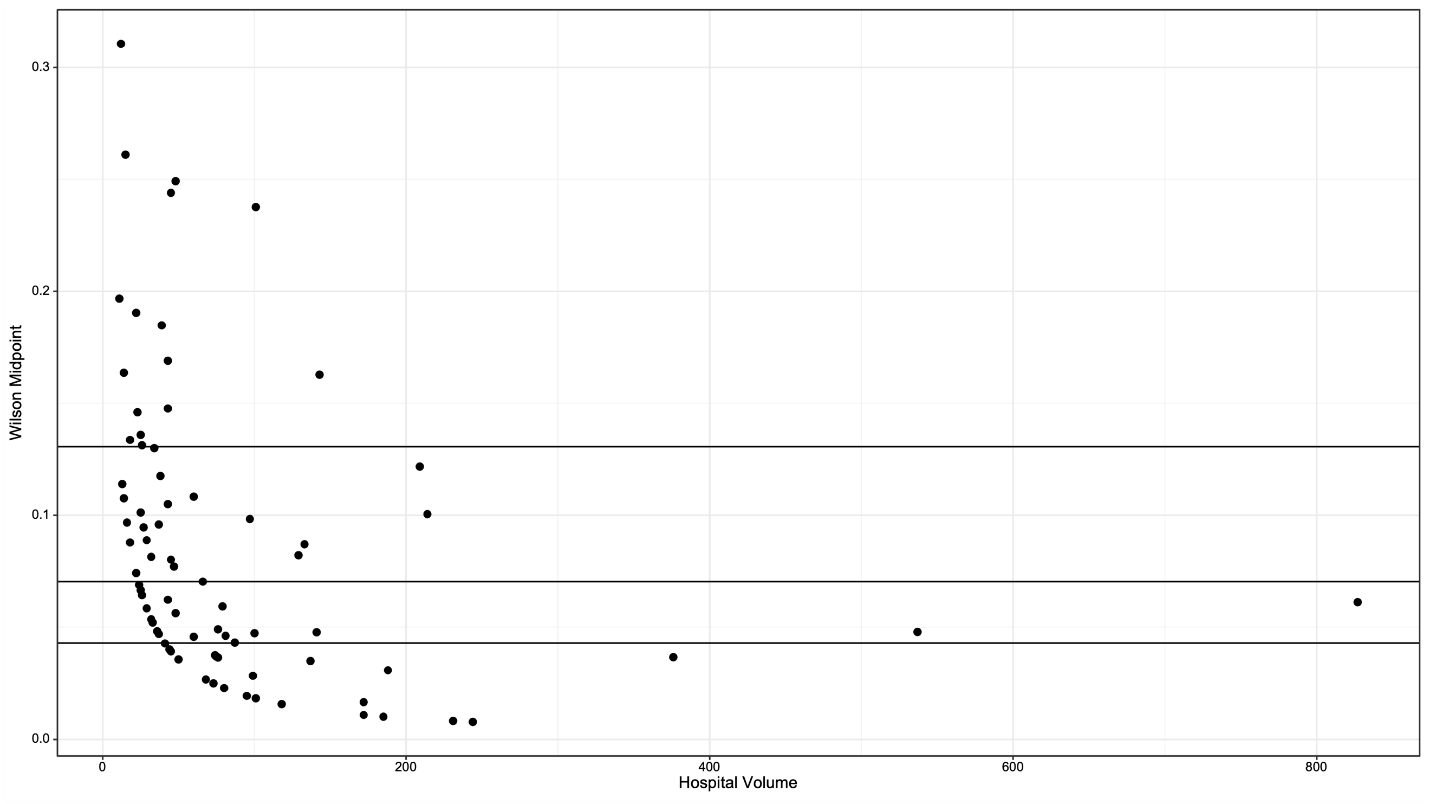
**

**Supplemental Figure 3: Relationship between Wilson Score Estimate and Facility Volume (A) and Serious Adverse Events (B) for all centers performing parathyroidectomies.** Red dots represent centers with <10 cases/year that were excluded from the main analysis of the report.

**
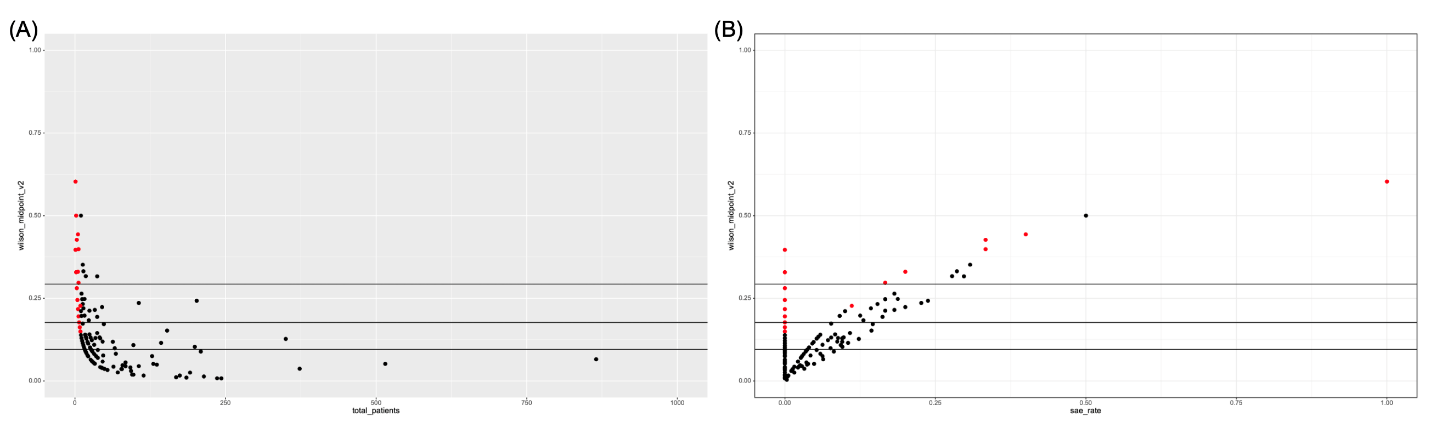
**
